# Adducin Is Involved in Endothelial Barrier Stabilization

**DOI:** 10.1371/journal.pone.0126213

**Published:** 2015-05-15

**Authors:** Daniela Kugelmann, Jens Waschke, Mariya Y. Radeva

**Affiliations:** Ludwig-Maximilians-University, Institute of Anatomy and Cell Biology, Department 1, München, Germany; Hungarian Academy of Sciences, HUNGARY

## Abstract

Adducins tightly regulate actin dynamics which is critical for endothelial barrier function. Adducins were reported to regulate epithelial junctional remodeling by controlling the assembly of actin filaments at areas of cell-cell contact. Here, we investigated the role of α-adducin for endothelial barrier regulation by using microvascular human dermal and myocardial murine endothelial cells. Parallel transendothelial electrical resistance (TER) measurements and immunofluorescence analysis revealed that siRNA-mediated adducin depletion impaired endothelial barrier formation and led to severe fragmentation of VE-cadherin immunostaining at cell-cell borders. To further test whether the peripheral localization of α-adducin is functionally linked with the integrity of endothelial adherens junctions, junctional remodeling was induced by a Ca^2+^-switch assay. Ca^2+^-depletion disturbed both linear vascular endothelial (VE)-cadherin and adducin location along cell junctions, whereas their localization was restored following Ca^2+^-repletion. Similar results were obtained for α-adducin phosphorylated at a site typical for PKA (pSer481). To verify that endothelial barrier properties and junction reorganization can be effectively modulated by altering Ca^2+^-concentration, TER measurements were performed. Thus, Ca^2+^-depletion drastically reduced TER, whereas Ca^2+^-repletion led to recovery of endothelial barrier properties resulting in increased TER. Interestingly, the Ca^2+^-dependent increase in TER was also significantly reduced after efficient α-adducin downregulation. Finally, we report that inflammatory mediator-induced endothelial barrier breakdown is associated with loss of α-adducin from the cell membrane. Taken together, our results indicate that α-adducin is involved in remodeling of endothelial adhesion junctions and thereby contributes to endothelial barrier regulation.

## Introduction

The vascular endothelium lining the inner surface of blood vessels precisely controls the passage of solutes, macromolecules, plasma proteins and inflammatory mediators and therefore provides a selective barrier between the blood and the surrounding tissue. Under inflammatory conditions, mainly in post-capillary venules, breakdown of the endothelial barrier function causes formation of intercellular gaps and enhanced vascular permeability. The latter results in severe subcutaneous and whole body cavity edema, which is the major risk factor for organ failure and death [1–4]. Therefore, our efforts are directed towards better understanding the mechanism underlying endothelial barrier integrity.

The endothelial barrier consists of two main types of intercellular junctions, i.e. tight junctions (TJs), sealing the intercellular cleft between neighboring cells, and the mechanical strength-providing adherens junctions (AJs). Those junctions are properly linked to the membrane-associated cortical actin cytoskeleton via their adaptor molecules and therefore may tightly control paracellular permeability [5]. Besides, the association of TJs and AJs with the actin cytoskeleton may explain why intracellular signaling regulating actin dynamics is critical for endothelial barrier function. In this line of thought, our previous study revealed that F-actin stabilization enhances barrier function whereas both depolymerization and hyperpolymerization of F-actin reduces endothelial barrier properties *in vitro* and *in vivo* [6]. The process of actin polymerization was shown to be tightly regulated by actin-binding proteins such as vasodilator-stimulated phosphoprotein (VASP) [7], cortactin [8] and adducins [9]. While the essential role of VASP and cortactin in regulation of endothelial barrier function has already been partly determined [3], to our best knowledge, the role of adducins in this process is largely unknown.

Adducins are a family of membrane skeletal proteins comprised of three closely related polypeptide isoforms encoded by distinct genes (α, β, and γ). While α- and γ-adducins are ubiquitously expressed in most tissues, the β isoform has a more restricted tissue distribution pattern [10]. As actin-binding proteins and key regulators of the actin polymerization process, adducins have been shown to play an essential role in maintenance of plasma membrane stability, cell-cell contact formation and regulation of cell motility [11]. In line with this, it was recently proposed that stable formation of AJs can be initiated by homophilic interaction between E-cadherins, followed by recruitment of ß-catenin and assembly of F-actin at epithelial cell-cell junctions. Once assembled at cell-cell junctions, F-actin recruits adducin which consequently links spectrin to F-actin and thus stabilizes the structure of cell-cell junctions [12]. Besides mediating spectrin-F-actin linkage, adducins are also reported to regulate actin filament bundling and capping. Additionally, it was recently demonstrated that downregulation of adducin attenuates formation of perijunctional F-actin bundles during reestablishment of epithelial AJs [13]. Moreover, a recent study from our group showed that silencing of adducin leads to considerable disruption of the actin cytoskeleton accompanied by impaired desmosome formation [14]. It was also shown that Rac1-deficient erythrocytes revealed defective association of the actin—spectrin scaffold and this was accompanied by changes in adducin phosphorylation [15]. In this respect, numerous reports demonstrated various functional consequences of adducin phosphorylation by different kinases. For instance, Rho-kinase phosphorylates adducin and thereby enhances its F-actin-binding activity *in vitro*. In contrast, protein kinase A (PKA)- and protein kinase C (PKC)-promoted adducin phosphorylation leads to opposite effects, i.e. reduced adducin binding activity to F-actin and to the F-actin-spectrin complex as well as to attenuation of spectrin recruitment to F-actin [16–18]. Adducin was also found to be rapidly removed from intercellular junctions and degraded in response to cytokines, invading bacteria and proapoptotic agents. Therefore, it was speculated that dysfunction of adducin can mediate disruption of epithelial barrier during infection and inflammation [13].

In the present study we examined the role of α-adducin in the formation and modulation of endothelial junctional integrity by using two different microvascular endothelial cell lines. Herein, we report that silencing of adducin not only negatively affects endothelial barrier formation but also modified Ca^2+^-dependent barrier reassembly. Moreover, we showed that in endothelial monolayers treatment with inflammatory mediators such as lipopolysaccharide (LPS), thrombin and tumor necrosis factor alpha (TNFα) resulted in loss of membrane-associated α-adducin.

## Materials and Methods

### Cell culture and antibodies used

Human Dermal Microvascular Endothelial Cells (HDMEC), the corresponding maintaining media and the Detach kit for further cell passaging were purchased either from PromoCell (Heidelberg, Germany) or from Lonza (Switzerland). The experiments were conducted with cells at passages 2 to 5 [19]. In addition, immortalized microvascular endothelial cells (MyEnd), previously isolated from murine myocardial tissue were used. MyEnd generation and characterization have been described elsewhere [20, 21]. Cells were grown in DMEM (Life Technologies, Karlsruhe, Germany), supplemented with Penicillin G/Streptomycin (50 U/ml) (Sigma-Aldrich, Munich, Germany) and 10% Fetal Calf Serum (FCS) (Biochrom, Berlin, Germany). Both cell types were cultured at 37°C in a humidified atmosphere of 5% CO_2_.

Vascular endothelial (VE)-cadherin in HDMEC was detected with goat anti-VE-cadherin pAb, purchased from Santa Cruz Biotechnology (Santa Cruz, U.S.A.). In MyEnd, VE-cadherin was immunolabeled with anti-VE-cadherin mAb (clone 11D4.1; undiluted hybridoma). For Western blot and immunofluorescence analyses in MyEnd cells, anti-α-adducin pAb (Abcam, Cambridge, United Kingdom) was applied. Primary anti-α-adducin mAb (Santa Cruz, Dallas, Texas, USA) was used for immunofluorescence staining in HDMEC. For visualization of α-adducin phosphorylated at Ser481, anti-α-adducin Ser481 mAb (Abcam, Cambridge, United Kingdom) was selected. Anti- γ-adducin (D11) mAb from Santa Cruz was used for immunoblot detection of γ-adducin. To observe proteins used as a loading control anti-α-tubulin mAb (Abcam, Cambridge, United Kingdom) and/or anti-ß-actin mAb obtained from Sigma-Aldrich (Munich, Germany) were utilized. Fluorescence monitoring of filamentous actin (F-actin) was achieved by using Alexa Fluor 488 phalloidin dye (Life Technologies, Karlsruhe, Germany). Nuclear morphology was controlled by direct labeling of the DNA with 4’,6-diamidino-2-phenylindole dihydrochloride (DAPI) (Roche Applied Science, Basel, Switzerland).

### Test reagents

Similarly to previous studies, an inflammatory mediator such as lipopolysaccharide (LPS) from *Escherichia coli* O111:B4 (Sigma-Aldrich, Munich, Germany, L2630) was used for 4 hours at 100 ng/ml. Human thrombin (Sigma-Aldrich, Germany) was applied at 10 U/ml for 5 min. Cell monolayers were incubated for 2 hours with 100 ng/ml of Tumor Necrosis Factor alpha (TNFα) (Biomol, Hamburg, Germany).

### Transfection with Small Interfering RNA (siRNA)

Significant transient down-regulation of α-adducin was achieved by adducin-specific ON-Target SMARTpool small interfering RNA (siRNA). Parallel transfection with a pool of ON-TARGET plus Non-Targeting (n.t.) siRNAs served as a negative control. The pools of siRNA sequences were obtained from Dharmacon, Waltham, U.S.A. To deliver the siRNA into the cell, TurboFect *in vitro* transfection reagent (Fermentas, St. Leonrot, Germany) was applied. The transfection was carried out according to the manufacturer’s instructions in cell monolayers at 70–80% confluency.

Permeability properties of transiently silenced MyEnd cell monolayers were monitored by TER-based measurements, which were started 24 or 48 hours after initial transfection and subsequent medium exchanged.

### Immunofluorescence analysis

HDMEC and MyEnd endothelial cells were grown to confluency on uncoated and gelatin-coated glass cover slips, respectively. As previously described [22], control and treated cell monolayers were fixed for 10 min at room temperature (RT) with freshly prepared 2% formaldehyde in PBS. Cells were washed with PBS and permeabilized with 0.1% Triton X-100 in PBS at RT. After rinsing with PBS, the adherent cell monolayer was blocked for 1 hour with 10% normal goat or donkey serum and 1% BSA in PBS. Incubation with primary antibodies (1:100 of each in PBS) was performed overnight at 4°C. After several washing steps with PBS, cell monolayers were treated with respective secondary antibodies conjugated with Cy3 or Cy2 (1 hour, RT). For visualization of filamentous actin, Alexa Fluor 488- conjugated—phalloidin was added to the secondary antibody at 1:100 dilution. Similarly, DAPI was applied to visualize nuclear morphological changes. Cell monolayers were photographed with a confocal microscope (Leica SP5, Mannheim and Wetzlar, Germany) equipped with a HCX PL APO Lambda blue 63× 1.4 oil immersion objective (Leica). The same microscope settings were used for all conditions tested.

### Protein extraction and Western blot analysis

Endothelial cells were grown to confluency. After quick washing with ice-cold PBS, cells were lysed with SDS-lysis buffer (25 mM HEPES, 2 mM EDTA, 25 mM NaF and 1% SDS) supplemented with protease inhibitor cocktail (Roche, Germany). Cells were scraped and the suspension was sonicated. Protein concentration was estimated using a BCA protein assay kit (Pierce/Thermo Scientific, Waltham, Germany). Lysates were subjected to gel electrophoresis and the blots were probed with the desired antibodies.

### Transendothelial electrical resistance (TER) measurement

To assess the endothelial barrier integrity of confluent cell monolayers, an ECIS Z Theta system (Applied Biophysics Inc, Troy, New York, U.S.A.) was used as previously described [22]. In brief, HDMEC and MyEnd cells were grown to confluency on uncoated and gelatin-coated gold microelectrodes 8W10E+ (Ibidi, Martinsried, Germany), respectively. Shortly before the experiment, the medium was exchanged and the microelectrodes were mounted onto the holders of the ECIS system, and incubated at 37°C and humidity of 5% CO_2_. All TER measurements were recorded at 4000 Hz, the experimentally determined optimal frequency for monitoring changes in the transednothelial resistance. After a short equilibration period of approximately 20 to 30 minutes, the baseline resistance was recorded.

### Calcium switch assay

For the calcium switch assay, endothelial cells were grown to confluency (vehicle) and then treated for 1 hour with 2.5 mM EGTA in a medium containing 50% of the supplements (Ca^2+^-depletion). In order to perform Ca^2+^-repletion, 5 mM CaCl_2_ was added for 8 hours.

### Data analysis

Data is expressed as mean ± SEM. Data analysis was carried out with Prism Software version 5 (Graph Pad, San Diego, California, U.S.A.). While 2-tailed paired Student’s t-test was used to compare two groups, One-way Analysis of Variance (ANOVA) followed by a Bonferroni's Multiple Comparison Test was applied to determine the differences among three groups. The results were considered statistically significant at p ≤ 0.05.

## Results

### α- and γ-adducin isoforms are abundant in microvascular endothelial cells

**Fig 1:**
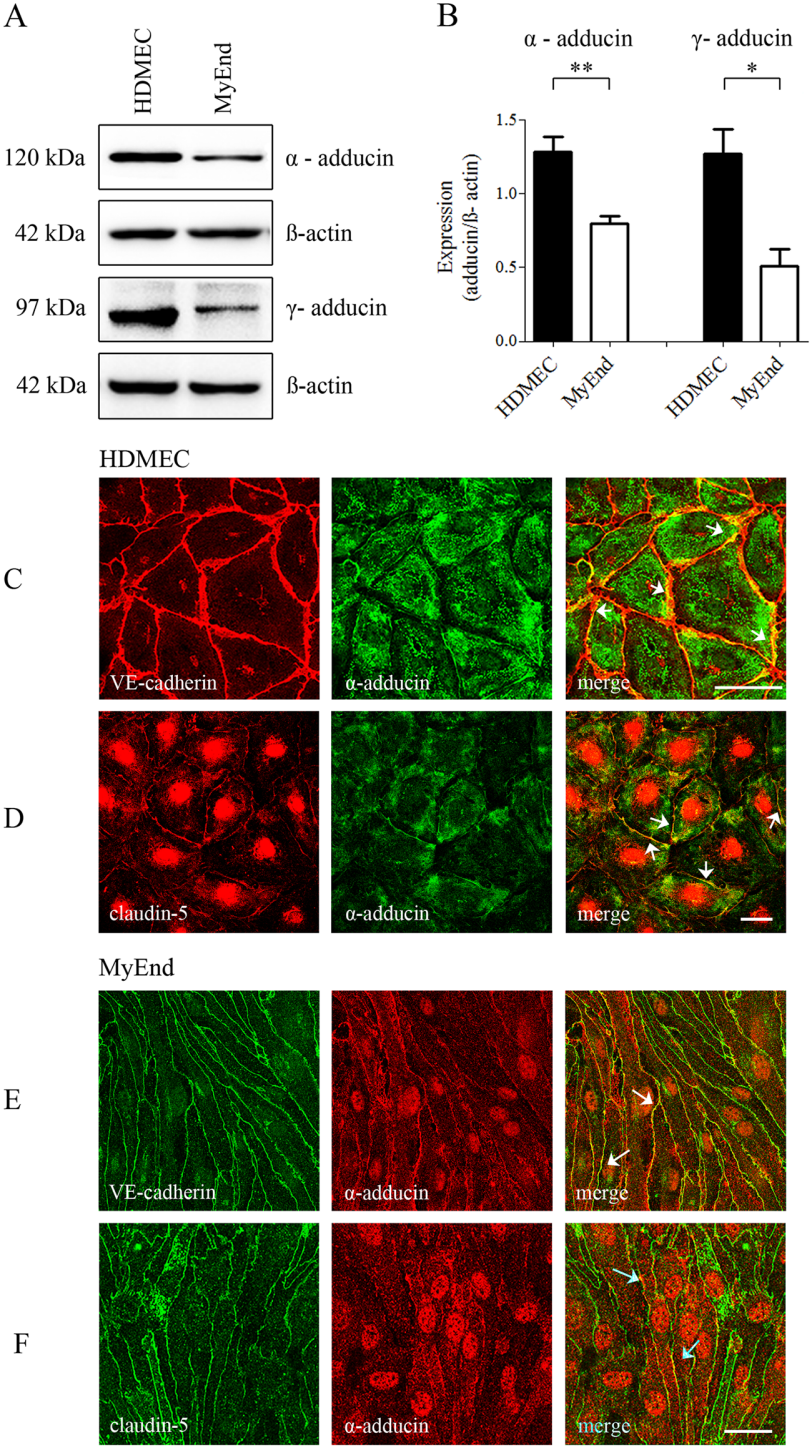
α- and γ-adducins are expressed in microvascular endothelium and α-adducin is colocalized with junctional proteins. (A) Protein abundance of α- and γ-adducins was evaluated by Western blot. α- tubulin or ß-actin were used to control for equal gel loading. (B) Expression profile of both adducin isoforms in bar graph representing the signal intensity analyzed by densitometry and normalized to the protein level of the respective loading control (Means ± SEM). In comparison to MyEnd cells, HDMEC cells showed significantly higher expression of α- and γ-adducins in total cell lysates (*P<0.05, **P<0.01, paired t-test, 2-tails) (C-F) Dual immunostaining for α-adducin with either AJ or TJ proteins in HDMEC and MyEnd cells revealed colocalization of α-adducin with VE-cadherin (AJ) and claudin-5 (TJ), respectively. The results are representative of three or more independent experiments. Scale bar = 20 μm.

The expression levels of α- and γ-adducin in endothelial cell models were examined by Western blot. Expression of both adducin isoforms was stronger in HDMEC than in MyEnd cells (Fig 1A and 1B). In both cell lines, α-adducin was detected at some segments of the cell membrane where it colocalized with VE-cadherin and claudin-5 under control conditions (Fig 1C and 1F, arrows). In HDMECs, cytoplasmic distribution was also observed whereas MyEnd cells displayed nuclear staining. Since the isotype controls and the secondary antibodies alone did not show pronounced unspecific staining of nuclei or cytoplasm, the observed α-adducin signals were most likely caused by the anti-α-adducin antibody (S1 and S2 Figs).

### α-adducin contributes to endothelial barrier formation by affecting adherens junction formation

To study the role of α-adducin in endothelial barrier formation and maintenance, down-regulation of endogenous α-adducin was induced by a siRNA approach applied in subconfluent MyEnd cells. Endothelial cells transfected with non-target (n.t.) siRNA were used as a control.

**Fig 2:**
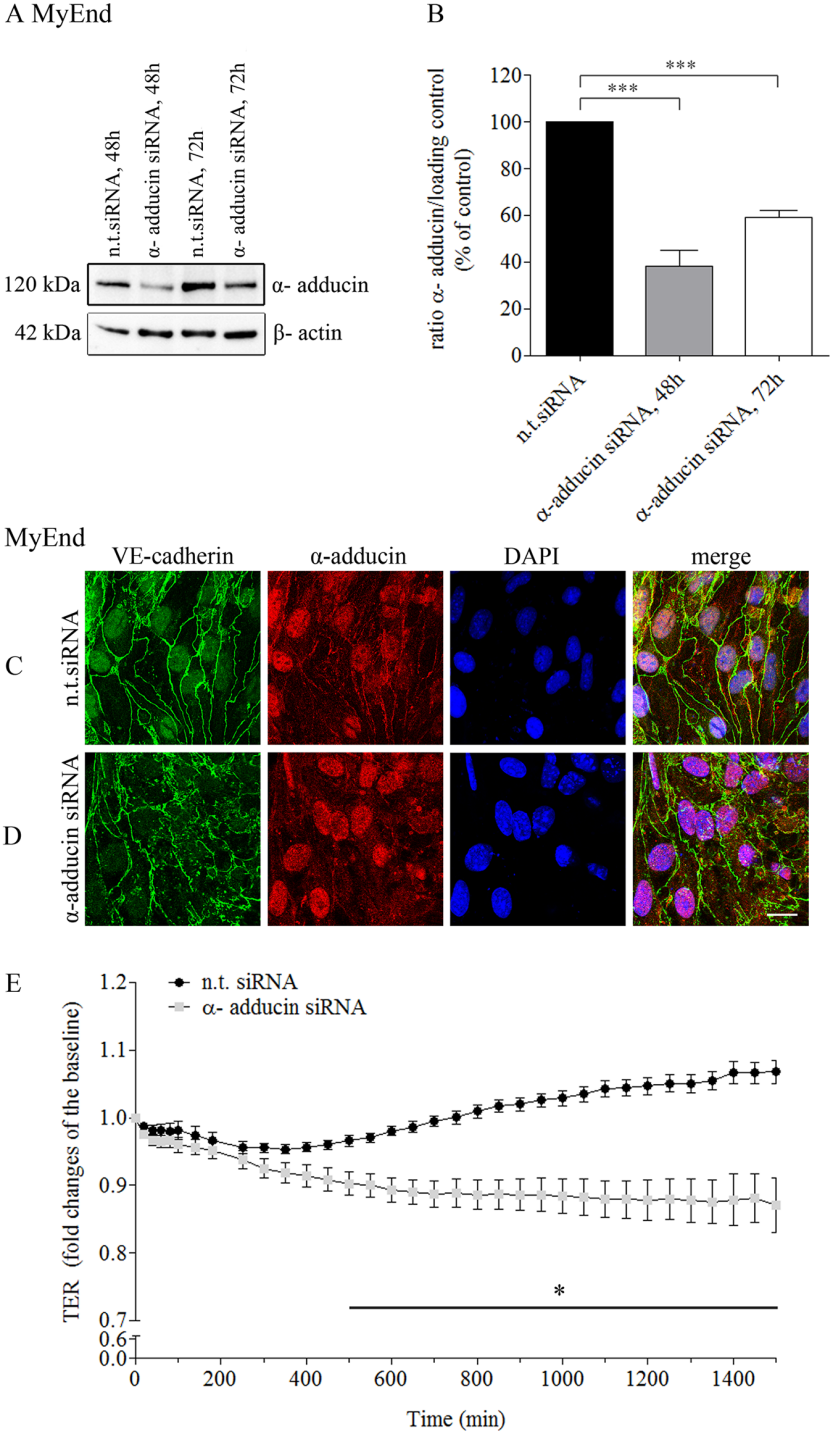
siRNA-mediated α-adducin knockdown significantly impaired endothelial barrier formation and caused loss of VE-cadherin from AJs. (A) Knockdown efficiency in MyEnd was confirmed by Western blot 48 hours and 72 hours after treatment with α-adducin-specific siRNAs. Cell monolayers exposed to non-target siRNA (n.t. siRNA) were used as a control. Equal gel loading was confirmed by α-tubulin or ß-actin (B) Bar graph shows densitomertic analysis of the respective blots, ***p≤ 0,0001, significance from control (n.t.siRNA), One-way.ANOVA. (C-D) Further evaluation of the depletion efficiency was achieved by immunofluorescence analysis. Double staining for simultaneous detection of α-adducin (red) and junctional VE-cadherin (green) was performed. (C) While significant α-adducin and VE-cadherin membrane staining was monitored under n.t. siRNA conditions, (D) target- specific knockdown led to a substantial reduction of α-adducin staining along cell borders. The effect was paralleled by severe disruption of VE-cadherin staining. Nuclei were visualized with DAPI (blue). Scale bar = 20 μm. (E) TER measurements in subconfluent, α-adducin depleted MyEnd cell monolayers revealed significant impairment of barrier formation 500 min after initial monitoring. Cells transfected with n.t. siRNA were used as a control. Data are representative of three or more independent experiments. *p ≤ 0.05 shows statistically significant difference between monolayers exposed to n.t and α-adducin specific siRNAs.

α-adducin downregulation was confirmed by Western blot analysis where significant decrease in the expression of α-adducin was demonstrated 48 hours after initial siRNA application. Depletion was less prominent 72 hours after siRNA-specific transfection (Fig 2A and 2B). Protein deficiency was further verified by immunofluorescence. Immunolabeling showed significant reduction of α-adducin staining along cell-cell contacts (Fig 2C and 2D). This was accompanied by considerable disorganization and fragmentation of VE-cadherin localization. No changes in nuclear morphology were found by DAPI staining (Fig 2C and 2D). These results were used to determine the time course for subsequent TER measurements. The latter were initiated one day after siRNA transfection and subsequent medium exchanged. TER measurements were run for at least 24 hours (Fig 2E) and the effect of α-adducin depletion on endothelial barrier formation and maintenance was monitored. In control monolayers TER increased with time and reached maximal values approximately 48 hours after transfection (1500 min: 106.7 ± 1.7% of baseline). In contrast, TER of α-adducin deficient cells did not increase (1500 min: 87.1 ± 4% of baseline).

### α-adducin localization at cell-cell junctions parallels integrity of adherens junctions (AJs)

**Fig 3:**
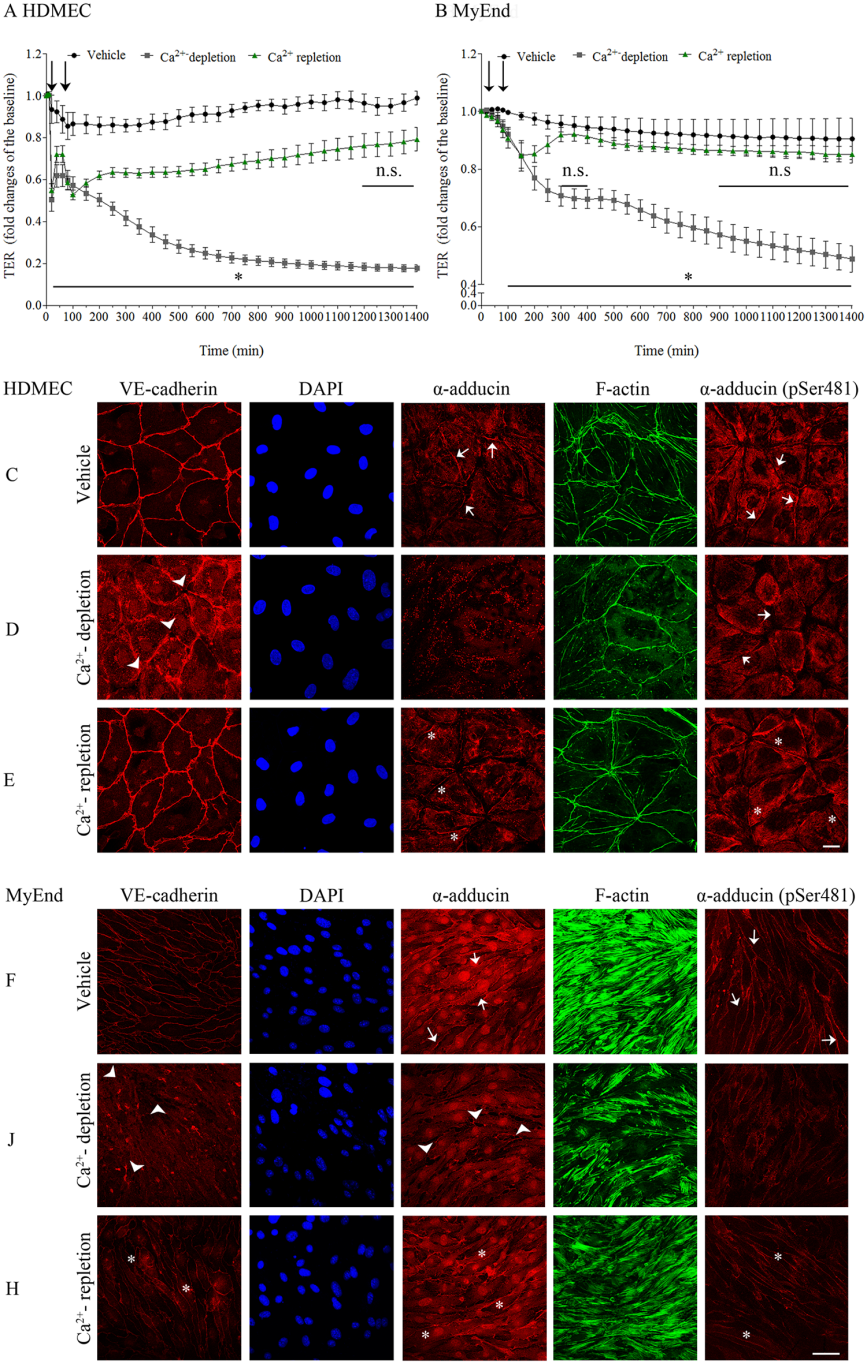
α-adducin accumulation along cell junctions paralleled AJ reorganization. Integrity and structural alterations in endothelial cell monolayers, subjected to a Ca^2+^-switch assay, were further evaluated either by TER measurements or by immunofuorescence analysis. (A-B) Time course of mean TER values showed that Ca^2+^-depletion (indicated by first arrow) induced a significant drop in TER which was largely recovered by Ca^2+^-repletion (second arrow) in HDMEC (A) and MyEnd (B) cells. *p ≤ 0.05 difference between control cells and cells depleted of Ca^2+^. Immunofluorescence analysis in HDMEC (C-E) and MyEnd cells (F-H) was performed. After subsequent fixation and permeabilization, cell monolayers were stained for VE-cadherin, α-adducin and α-adducin (pSer481). Additionally, Alexa Fluor 488 phalloidin was used for staining of F-actin. To identify the nuclei, cells were counterstained for DAPI (blue). (C and F) Immunofluorescence in HDMEC (C) and MyEnd cells (F) revealed that under control condition, in parallel to VE-cadherin, α-adducin and α-adducin (pSer481) are markedly localized along cell-cell borders (arrows). (D and J) In both cell lines Ca^2+^-depletion led to irregular (arrows) or absent VE-cadherin staining, associated with reduced localization or complete absence of α-adducin and α-adducin (pSer481) at cell-cell-junctions. This was accompanied with reduced staining of F-actin and induced intercellular gap formation (arrowhead). (E and H) In contrast, repletion of Ca^2+^ restored linear VE-cadherin staining as well as accumulation of α-adducin and α-adducin (pSer481) at cell-cell borders (stars). The staining of F-actin was increased as well. For all conditions, no morphological changes in the nuclei were observed. Images are representative of three or more independent experiments. Scale bar = 20 μm.

Recent studies revealed that adducins are localized at epithelial cell-cell junctions where they specifically colocalize with junctional E-cadherin [12, 23]. In endothelial cells, vascular endothelial (VE)-cadherin is the main adhesive molecule of intercellular AJs. VE-cadherin mediates Ca^2+^-dependent homophilic interaction responsible for adhesion of endothelial cells and formation of a confluent monolayer and the endothelial barrier [24]. Therefore, to further test whether the peripheral localization of α-adducin is linked with integrity of VE-cadherin-based AJs and endothelial barrier function, endothelial junctional remodeling was induced by a Ca^2+^ switch assay. The latter involves removal of extracellular Ca^2+^ (Ca^2+^-depletion) to trigger disassembly of already formed AJs. In contrast, subsequent Ca^2+^ re-addition (Ca^2+^-repletion) induces recovery of junctional structure and function [25]. Thus, in order to test whether the junction reorganization in response to Ca^2+^ switch can effectively modulate endothelial barrier properties, TER measurements in HDMEC (Fig 3A) and MyEnd cells (Fig 3B) were performed. For that purpose, microvascular endothelial cells were grown to confluency in medium containing 100% of supplements. During Ca^2+^-depletion, cells were treated with 2.5 mM EGTA for 1 hour in medium with 50% supplements (indicated by first arrow in Fig 3A and 3B). Ca^2+^-repletion was performed by re-addition of CaCl_2_ to a final concentration of 5 mM for 8 hours (second arrow in Fig 3A and 3B). The analysis revealed that as a result of Ca^2+^-depletion TER was drastically reduced in both cell lines, whereas Ca^2+^-repletion led to recovery of endothelial barrier properties with different time courses. In MyEnd cells TER values were similar to controls from 300 min on and were not significantly different after 900 min (Fig 3B). In HDMEC monolayers barrier reformation was slower (Fig 3A), which may be caused by the more prominent decrease in TER immediately after EGTA application (Ca^2+^-depletion).

Furthermore, HDMEC and MyEnd cell monolayers subjected to Ca^2+^-switch were immunostained for VE-cadherin and α-adducin as well as labeled for F- actin using Alexa 488-phalloidin. In order to verify normal nuclear morphology, nuclei were stained with DAPI. Moreover, since α-adducin is a substrate of protein kinase A (PKA), a molecule implicated in cAMP-mediated endothelial barrier stabilization [26], endothelial cell monolayers were co-stained for α-adducin phosphorylated at a site typical for PKA (α-adducin pSer481). Under control conditions the continuous VE-cadherin distributions was paralleled with pronounced membrane localization of α-adducin. Distribution of α-adducin pSer481 resembled the pattern of α-adducin as described above with the only difference that in MyEnd cells almost all cells displayed membrane localization (Fig 3C and 3F, arrows). Depletion of Ca^2+^ resulted in severe VE-cadherin dissociation from AJs, accompanied by pronounced disruption of α-adducin and α-adducin pSer481-localization along the cell periphery (Fig 3D and 3J). Additionally, reduction of F-actin staining and considerably increased gap formation was observed (Fig 3D and 3J, arrowheads). However, replacement of Ca^2+^ not only increased the staining of intracellular F-actin, but also restored the distribution of VE-cadherin, α-adducin and α-adducin pSer481 at cell-cell borders (Fig 3E–3H, stars). In contrast, DAPI staining revealed no morphological changes of nuclei under all conditions.

### Depletion of α-adducin impaired Ca^2+^-dependent endothelial junction re-assembly

**Fig 4:**
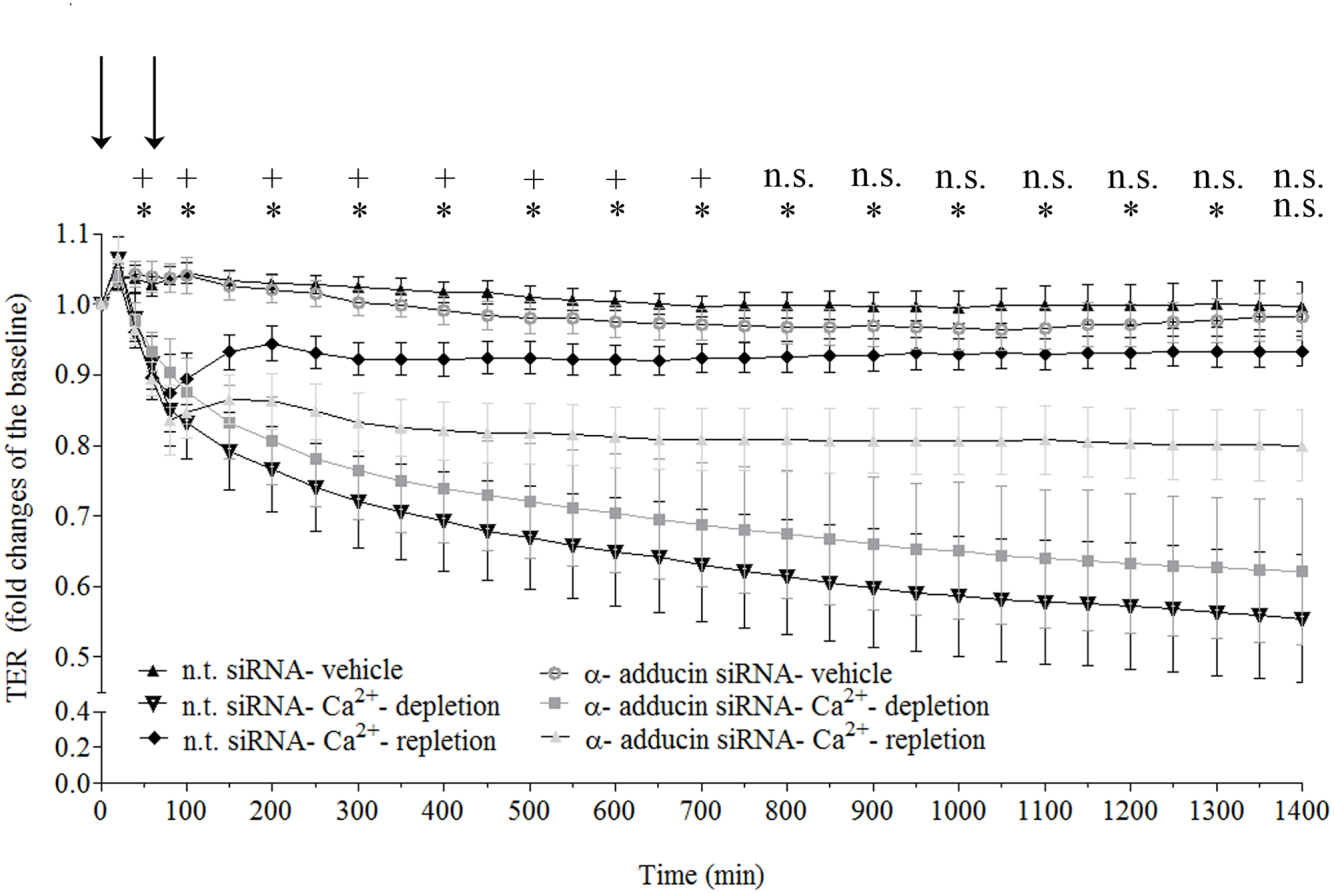
Depletion of α-adducin attenuates Ca^2+^-mediated recovery of junctional integrity. Endothelial barrier integrity of monolayers transiently transfected with α-adducin siRNA and subjected to Ca^2+^-switch assay was examined by TER. Cells treated with n.t siRNA were used as control. Time course of mean TER values showed that endothelial barrier recovery was significantly attenuated in adducin-depleted monolayers re-exposed to Ca^2+^ (Ca^2+^-repletion) when compared to control monolayers transfected either with n.t. or adducin-specific siRNA. In parallel, treated with n.t. siRNA and re-exposed to extracellular Ca^2+^ monolayers, displayed resistance quite similar to vehicle monolayers. Data were collected from three independent experiments. *p ≤ 0.05 indicates difference between TER monitored in α-adducin siRNA-Ca ^2+^-repletion and n.t siRNA-vehicle. +p ≤ 0.05 indicates difference between TER monitored in α-adducin siRNA- Ca ^2+^-repletion and α-adducin siRNA-vehicle; n.s.- denotes p ≥ 0.05.

We next analyzed whether α-adducin is essential for reestablishment of Ca^2+^-dependent endothelial barrier integrity by using Ca^2+^-switch model in cell monolayers depleted for α-adducin for 48 hours as described above (Fig 4). In comparison to control monolayers, adducin-depleted cells showed a significant and stable reduction in TER when re-exposed to extracellular Ca^2+^. No significant differences in TER were monitored in Ca^2+^- depleted monolayers transfected with either n.t or adducin-specific siRNA. Moreover, cells transfected with n.t. siRNA recovered following Ca^2+^- repletion and were not statistically different from untreated monolayers. In contrast, Ca^2+^-repleted, adducin-deficient monolayers did not completely recover barrier function. Taken together, these results confirm the prominent role of α-adducin in the modulation of Ca^2+^-dependent endothelial barrier reorganization.

### Endothelial barrier breakdown induced by inflammatory mediators is paralleled with remarkable reduction of α-adducin and α-adducin (pSer481) at cell junctions

**Fig 5:**
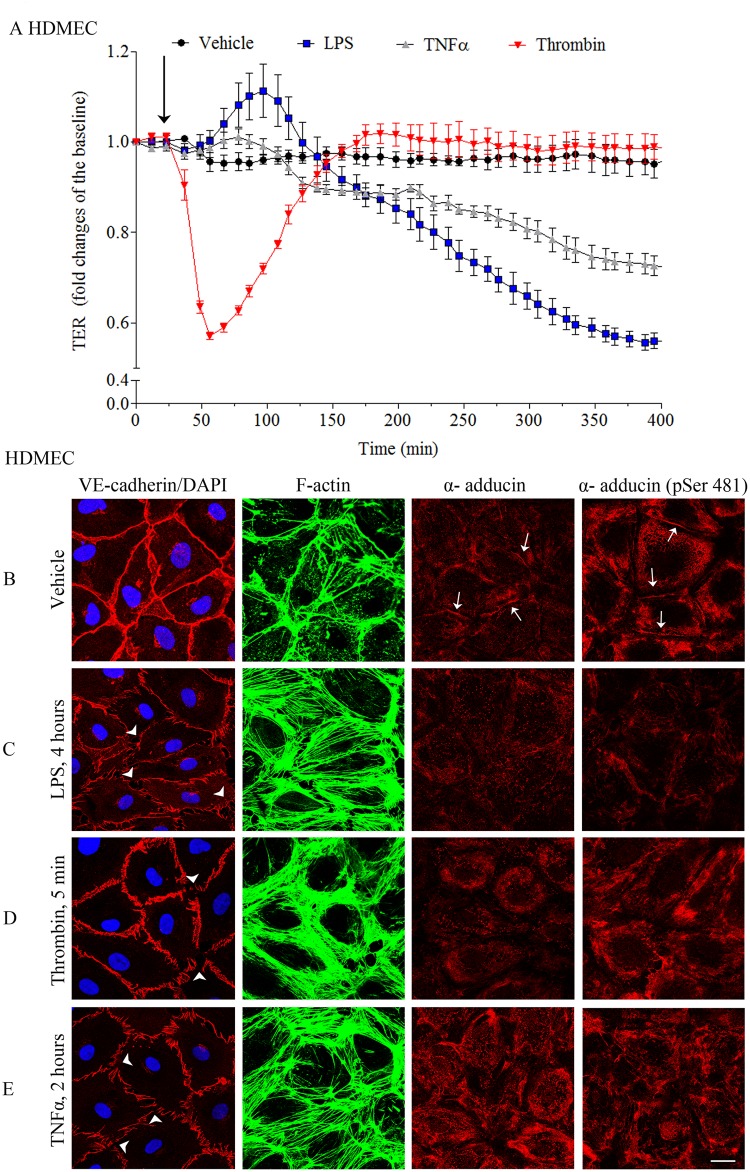
Changes in adducin localization parallel to endothelial barrier dysfunction induced by inflammatory mediators. Barrier integrity of HDMEC control monolayers and monolayers exposed to different inflammatory mediators such as thrombin, LPS and TNFα was evaluated by TER measurements and immunofluorescence analysis. (A) TER in control cells or HDMEC treated with inflammatory agents. Arrows denote the application of mediators. Results are expressed as a mean ± SEM (n = 2). Mediator-induced decrease in TER was accompanied by alterations in the immunostaining pattern not only of the adhesion junctional protein VE-cadherin but also of α-adducin and α-adducin (pSer481). (B-E) HDMEC monolayers exposed to inflammatory mediators or control cells were immunostained for VE-cadherin, α-adducin and α-adducin (pSer481). Staining for F-actin and for nuclei was also accomplished with Alexa Fluor 488 and DAPI, respectively. In comparison to control condition (B) where VE-cadherin was linearly distributed along cell borders and α-adducin as well as α-adducin (pSer481) were present at cell-cell contacts (arrows, B), treatment with LPS (C), thrombin (D) and TNFα (E) led to considerable intercellular gap formation (arrowheads, C-E), paralleled by pronounced increased stress fiber formation and fragmented VE-cadherin distribution. This effect was associated with almost complete loss of α-adducin membrane staining. No differences in the nuclear morphology were observed under these conditions. Scale bar = 20 μm.

Finally, we sought whether the localization of α-adducin at cell junctions also parallels endothelial barrier dysfunction under inflammatory conditions. Thus, HDMEC monolayers were treated with different barrier-disruptive mediators such as LPS (4 hours), thrombin (5 min) and TNFα (2 hours). As previously published [19, 27, 28], TER measurements of mediator-treated HDMEC confirmed the effective endothelial barrier breakdown induced by LPS, thrombin and TNFα (Fig 5A). Subsequent immunostaining for VE-cadherin, α-adducin and α-adducin (pSer481) as well as direct staining of F-actin and DAPI was performed. Under control conditions, VE-cadherin and both adducin forms were distributed at cell borders as described above. Untreated monolayers also displayed relatively few stress fibers visualized by F-actin staining (Fig 5B). In contrast, drastically increased stress fiber formation, paralleled with severe VE-cadherin interdigitation and pronounced gap formation (arrowheads) was monitored under LPS, thrombin or TNFα- treatment. These morphological changes were associated with significant loss of both α-adducin and α-adducin (pSer481) junctional staining (Fig 5C–5E). Nuclear staining with DAPI demonstrated intact cell nuclei under all conditions (Fig 5B–5E).

## Discussion

Besides being key regulators of actin polymerization process, adducins are also reported to control cell migration and cell-cell-contact formation [9, 29]. In respect to the latter, several studies reported the association of adducins with intercellular contacts in epithelia [9, 23, 30]. More specifically, a study of Naidenov *et al*., demonstrated the role of α- and γ- adducin isoforms in the modulation of tight and adhesion junctions [13]. It was reported recently that adducin is crucial for keratinocyte cohesion [14], which is in line with data suggesting that α-adducin depletion impairs E-cadherin- mediated adhesion of MDCK cells [12]. However, although the study of Cappuzzello *et al*., demonstrated that α-adducin contributes to the regulation of endothelial function in hypertension *in vitro* and *in vivo* [31], hardly anything is known about the role of adducins in formation and maintenance of endothelial barrier integrity. Thus, our study provides the first evidence that α-adducin is implicated in formation of endothelial barrier integrity and is involved in modulation of Ca^2+^-dependent AJ reorganization. Similar to the study of Chen and co-workers [12] we demonstrated that prevention of VE-cadherin homophylic interaction by Ca^2+^-depletion impairs the recruitment of α-adducin to cell-cell contacts. In contrast, Ca^2+^readdition restored both VE-cadherin homophylic interaction and accumulation of α-adducin and α-adducin (pSer481) at cell-cell-junction, suggesting that the recruitment of α-adducin to the junctions as well as its phosphorylation status may also be important for the stability of VE- cadherin interaction.

Adducins were reported to be substrates of PKA, PKC and Rho kinase [16–18, 29], however the role of adducin phosphorylation in endothelial cells is not clear yet. While cAMP/PKA-mediated signaling is well known to strength endothelial function [32, 33], other studies in non-endothelial tissues showed that PKA- and PKC-promoted adducin phosphorylation leads to reduced adducin binding to F-actin and to the F-actin-spectrin complex as well as promotes attenuation of spectrin recruitment to F-actin [16–18], which was shown to contribute to enchanced remodeling of the cortical cytoskeleton and destabilization of cell-cell contacts [25]. Since our previous studies indicate that cAMP-mediated signaling is impaired under inflammatory conditions [19, 27, 28], we investigated whether junctional localization of adducin (pSer481) is affected by inflammatory mediators. Similarly to the Ca^2+^-switch assay, we found that endothelial barrier breakdown induced by LPS, thrombin or TNFα was associated with loss of α-adducin and α-adducin (pSer481) from cell junctions. However, more detailed investigations are necessary to provide deeper insight into the role of adducin phosphorylation in endothelial barrier regulation.

Furthermore, although studies on α-adducin-null mice did not report specific abnormalities in vascular integrity, the dynamic role for adducin in assembly, maintenance and regulation of cell membranes was suggested [34, 35]. In this respect, the most important outcome of our study is that α-adducin is required for endothelial barrier formation. Monolayers transiently transfected with α-adducin siRNA exhibited attenuated formation of the endothelial barrier. The same holds true for more rapid barrier reassembly, since we could show that depletion of α-adducin led to impaired reestablishment of the endothelial barrier when monolayers were subjected to Ca^2+^-switch. Our observation confirmed previously published data in colonic epithelial cells demonstrating a significant delay in TER recovery in adducin-depleted cell monolayers subjected to Ca^2+^-switch [13]. Given the fact that adducins are associated with and participate in the regulation of different cytoskeletal structures, we suggest that they may regulate endothelial junctions also by controlling either organization of the spectrin lattice or assembly of actin filaments in areas of cell-cell contacts, as was shown in epithelial cells [12, 13]. In this regard, similar to recently published report [14], we observed that adducin silencing induced disruption of the actin cytoskeleton (S3 Fig). Moreover, we found that F-actin staining was significantly reduced when VE-cadherin homophilic binding was disturbed by Ca^2+^-depletion, an effect that was also paralleled by reduced adducin staining. Therefore, further examinations should be performed to elucidate whether VE-cadherin binding regulates actin dynamic via adducins.

In conclusion, our study reveals a novel role of α-adducin in the regulation of endothelial barrier integrity which at least in part may be due to α-adducin-mediated control of AJ assembly.

## Supporting Information

S1 Fig Isotype specificity of the primary antibody. To test the isotype specificity of the primary antibody, HDMEC and MyEnd cell monolayers, handled identically as controls, were simultaneously immunostained with normal mouse and rabbit IgG. The latter represented the same subclasses of α-adducin Abs which have been used in the study. DAPI staining was used to confirm confluent cell monolayers. Besides the slight cytoplasmic staining of rabbit IgG-Cy3 in MyEnd cells, no unspecific immunosignals were detected. Scale bar = 20 μm.
(TIF)

S2 Fig Cy-labeled antibodies are specific. To test the specificity of the secondary antibody, different cyanine dyes (Cy)- conjugated secondary antibodies (Abs) were applied to cell monolayers grown under control conditions. In order to confirm confluency of the cell monolayer, nuclei were stained directly with DAPI. None of the immunolabelings showed unspecific for the secondary antibody staining, which indicates that the Cy-label antibodies are specific to the respective primary antibody. Scale bar = 20 μm. dag is donkey anti-goat; garb is goat anti-rabbit; gam is goat anti-mouse; gart is goat anti-rat.
(TIF)

S3 Fig Adducin silencing induced disruption of the actin cytoskeleton. MyEnd monolayers transfected with n.t siRNA and adducin-specific siRNAs were stained for α-adducin and F-actin. (A) Under control conditions, α-adducin localized partly along cell junctions which was accompanied with intensive F-actin staining all over the cells. (B) In contrast, α-adducin-depleted monolayers showed reduced adducin staining at cell junctions paralleled by significantly attenuated staining for F-actin. Scale bar = 20 μm.
(TIF)
