# Supplementary figures and images for: Adducin Is Involved in Endothelial Barrier Stabilization
Source: PLoS One. 2015 May 15;10(5):e0126213. doi: 10.1371/journal.pone.0126213 (PMC4433183; doi:10.1371/journal.pone.0126213)

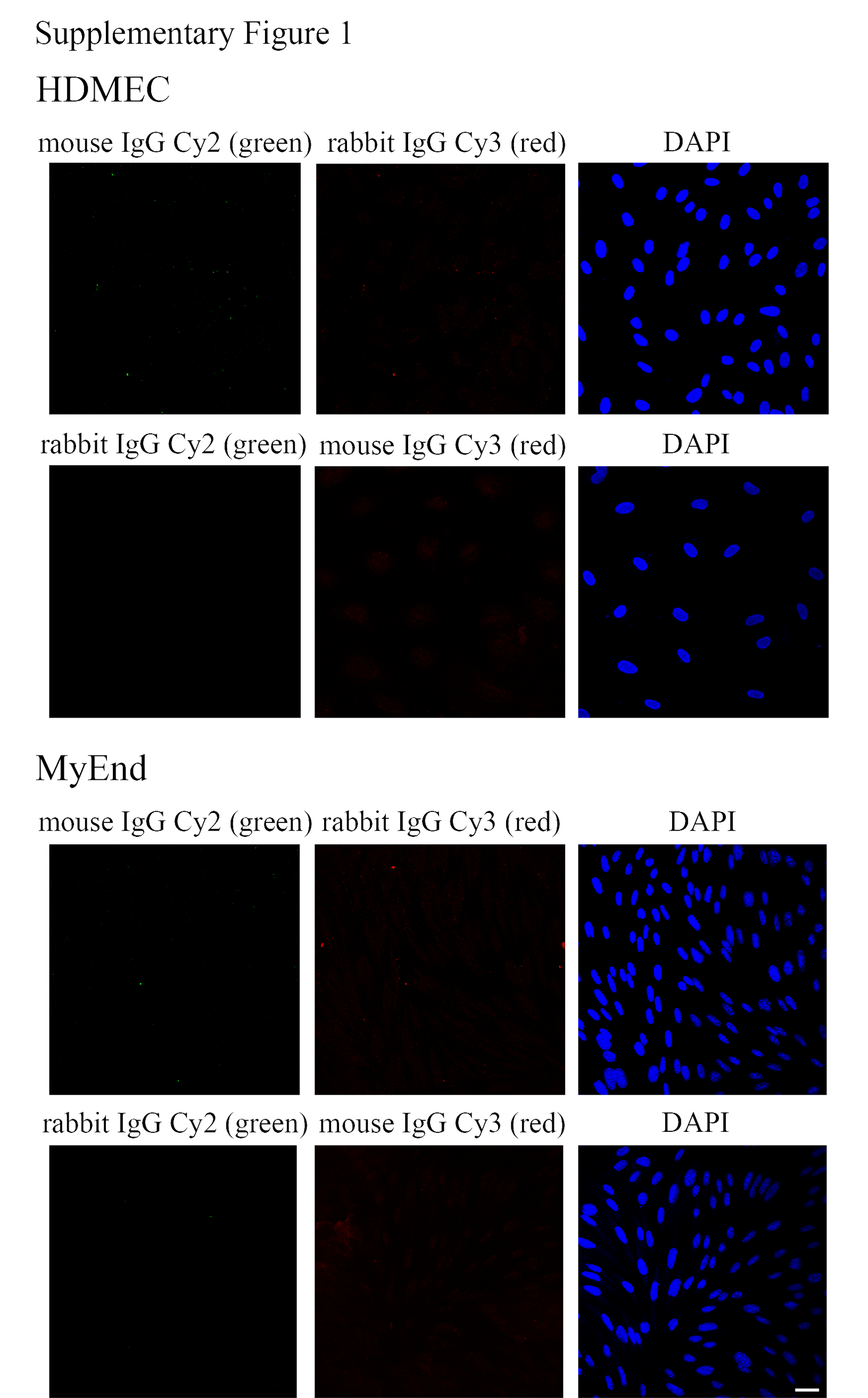

Supplement: S1 Fig — To test the isotype specificity of the primary antibody, HDMEC and MyEnd cell monolayers, handled identically as controls, were simultaneously immunostained with normal mouse and rabbit IgG. The latter represented the same subclasses of α-adducin Abs which have been used in the study. DAPI staining was used to confirm confluent cell monolayers. Besides the slight cytoplasmic staining of rabbit IgG-Cy3 in MyEnd cells, no unspecific immunosignals were detected. Scale bar = 20 μm. (TIF) [file pone.0126213.s001.tif]

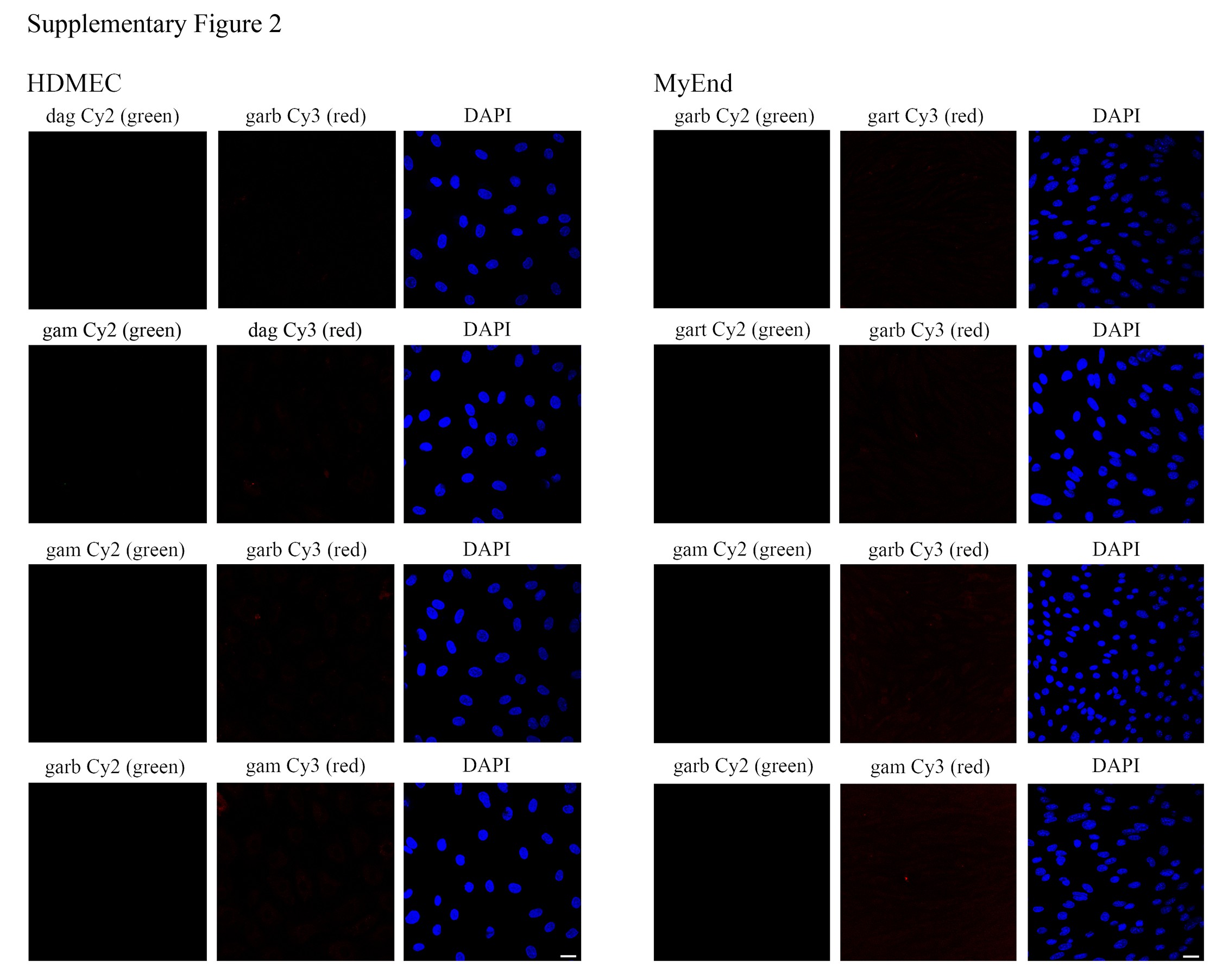

Supplement: S2 Fig — To test the specificity of the secondary antibody, different cyanine dyes (Cy)- conjugated secondary antibodies (Abs) were applied to cell monolayers grown under control conditions. In order to confirm confluency of the cell monolayer, nuclei were stained directly with DAPI. None of the immunolabelings showed unspecific for the secondary antibody staining, which indicates that the Cy-label antibodies are specific to the respective primary antibody. Scale bar = 20 μm. dag is donkey anti-goat; garb is goat anti-rabbit; gam is goat anti-mouse; gart is goat anti-rat. (TIF) [file pone.0126213.s002.tif]

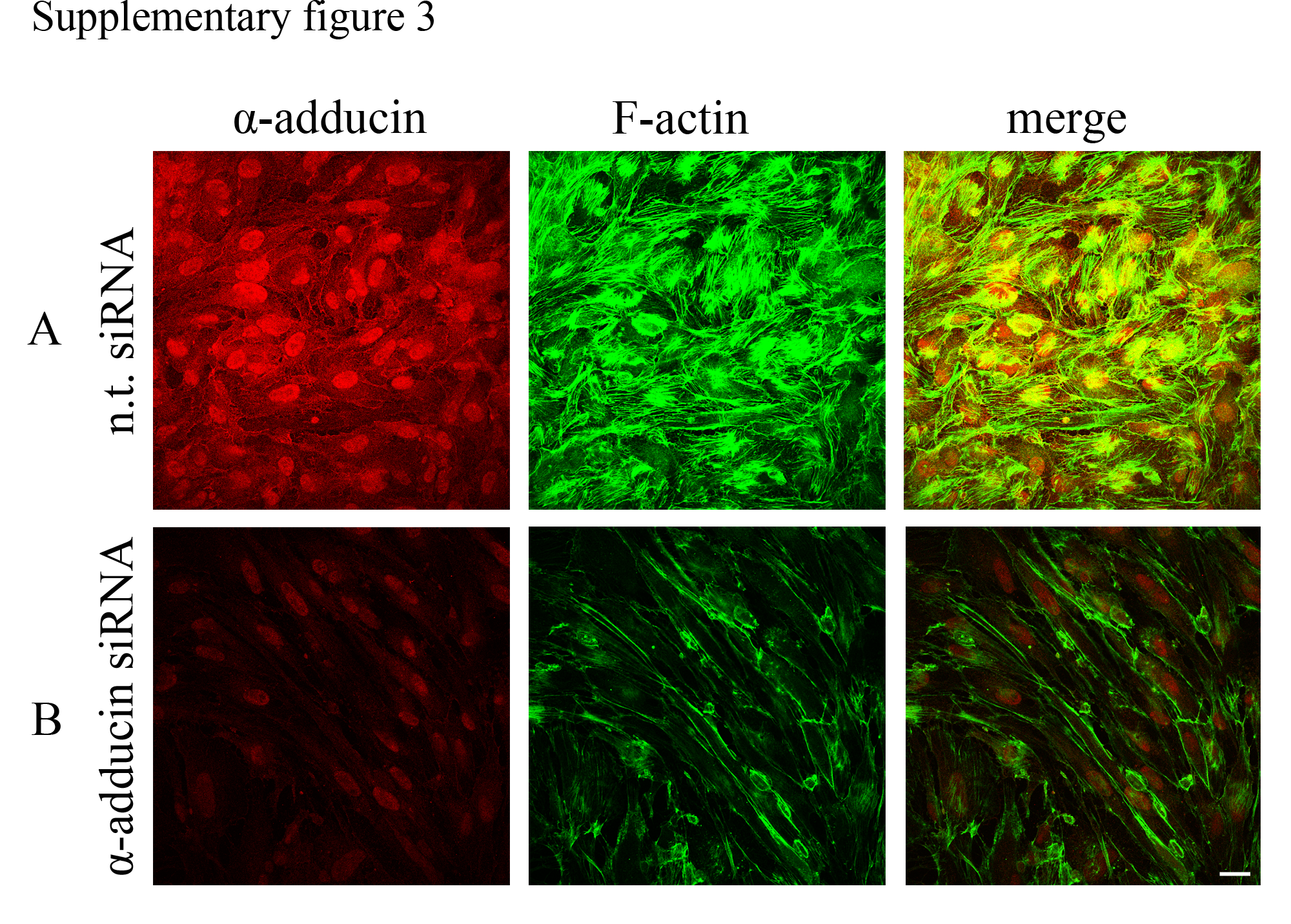

Supplement: S3 Fig — MyEnd monolayers transfected with n.t siRNA and adducin-specific siRNAs were stained for α-adducin and F-actin. (A) Under control conditions, α-adducin localized partly along cell junctions which was accompanied with intensive F-actin staining all over the cells. (B) In contrast, α-adducin-depleted monolayers showed reduced adducin staining at cell junctions paralleled by significantly attenuated staining for F-actin. Scale bar = 20 μm. (TIF) [file pone.0126213.s003.tif]
